# Interspecies comparison of simultaneous thrombin and plasmin generation

**DOI:** 10.1038/s41598-020-60436-1

**Published:** 2020-03-03

**Authors:** Ivan D. Tarandovskiy, Hye Kyung H. Shin, Jin Hyen Baek, Elena Karnaukhova, Paul W. Buehler

**Affiliations:** 1 Laboratory of Biochemistry and Vascular Biology, Center for Biologics Evaluation and Research, Food and Drug Administration, 10903 New Hampshire Avenue, Silver Spring, Maryland 20993 USA; 2 Department of Pathology, The University of Maryland School of Medicine, 670 W. Baltimore Street, Baltimore, Maryland 21201 USA; 3 The Center for Blood Oxygen Transport and Hemostasis, Department of Pediatrics, The University of Maryland School of Medicine, 670 W. Baltimore Street, Baltimore, Maryland 21201 USA

**Keywords:** Proteases, Animal disease models, Cardiovascular models, Spectrophotometry, Cardiovascular biology

## Abstract

Animal models of hemostasis are often extrapolated to humans; however, only a few studies have compared coagulation and fibrinolysis across species. Simultaneous thrombin (TG) and plasmin (PG) generation is useful to assessing coagulation and fibrinolysis within the same sample. In this study, we performed simultaneous TG and PG analysis in blood plasma samples from humans and 6 species commonly evaluated in pre-clinical research. TG and PG were investigated in male and female donor platelet-poor plasmas (PPP) obtained from 28 healthy humans, 10 baboons, 12 rhesus monkeys, 20 Yorkshire pigs, 20 Sprague-Dawley rats, 10 New Zealand White rabbits and 14 Hartley guinea pigs. The continuous generation of the 7-amino-4-methylcoumarin (AMC) from substrates specific to thrombin or plasmin was monitored. The thrombin and plasmin concentration peak heights (PH) and production rates (PR) were calculated. TG and PG parameters from baboon and rhesus macaque plasma approximated that of humans. The other species differed significantly from both human and non-human primates. For example, swine and rat plasmas demonstrated similar TG, but swine plasmas did not generate plasmin. TG and PG parameters from Guinea pig samples were extremely low, while rabbit plasmas showed variable PG curves demonstrating one or two peaks with low and high PR values, respectively. Correlations between PH and PR values were significant with the exceptions of human PG, baboon TG, rat TG and Guinea pig PG. These findings are informative to pre-clinical animal species selection and optimization of coagulation and fibrinolysis translational research.

## Introduction

Animal models of hemostasis and thrombosis are widely used in basic and pharmaceutical research^1^. Results are frequently extrapolated to humans; however, data obtained from animal models often falls short of accurate predictions in human response^2^. Further, assays have limited relevant comparisons of hemostasis in human blood with that of differing animal species. The novel global assays of hemostasis such as thrombin generation (TG) and thromboelastography (TEG) or thromboelastometry (ROTEM) provide an opportunity to assess similarities in coagulation and fibrinolysis *ex vivo* on blood obtained from different species. Although TG and thromboelastographic assays are often used for analysis of hemostasis in different animals^1,3–9^, comparison among different species is rarely made. There are limited studies that compare blood coagulation and fibrinolysis in various animals under the same conditions^10–17^. TG assays are sensitive for use in clinical and basic research studies; however, fibrinolysis testing primarily focused on clot lysis time, is often insufficiently sensitive and demonstrates high data variability^8,9,11^. Since 2011, several hemostasis assays have focused on simultaneous registration of thrombin and plasmin generation (PG) in plasma using tissue factor (TF) and tissue plasminogen activator (tPA), respectively^18–21^. These approaches suggest high sensitivity to assessment of coagulation and fibrinolysis and to the impairment of these systems^21–25^. PG is a novel assay, and until the present study it has not been applied to interspecies comparisons of fibrinolysis. Here we performed a simultaneous TG and PG assay (STPGA) to compare human response to a range of species (baboon, Rhesus macaque, swine, rat, rabbit and guinea pig) that are widely evaluated as animal models in hemostasis research and therapeutics development.

## Results

### STPGA curves ranges

**Figure 1:**
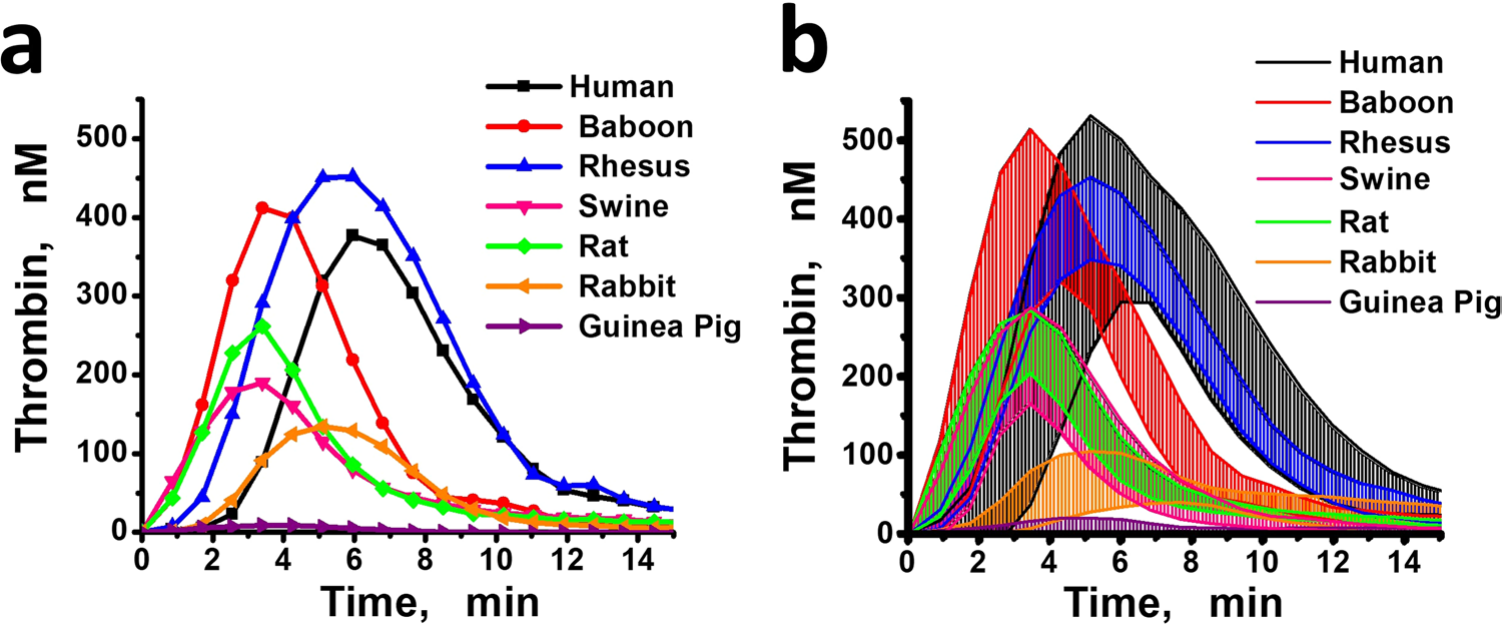
(**a**) The representative TG curves from each species. (**b**) The range of thrombin generation curves for PPPs from different species: the mean thrombin concentrations ± SD for each moment of time. The lower line for each species represents mean-SD between all the samples in the group, and the upper line represents mean + SD.

**Figure 2:**
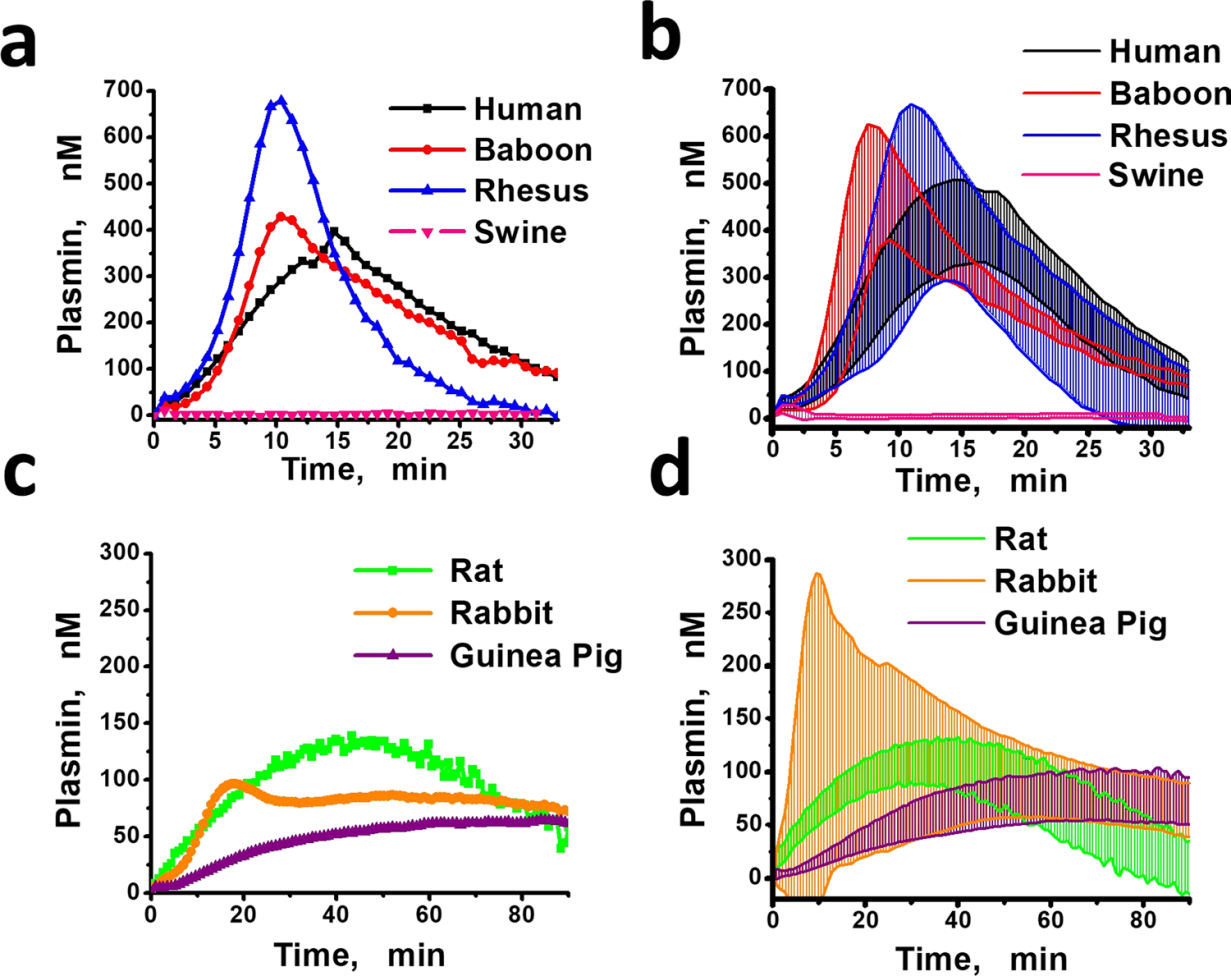
The representative PG curves and the ranges of plasmin generation curves for platelet-poor plasmas (PPP) from different animals: mean plasmin concentrations ± SD for each moment of time. The lower line for each species represents mean-SD between all the samples in the group, and the upper one represents mean + SD.

Figures 1 and 2 demonstrate the ranges of TG and PG curves (mean thrombin and plasmin concentrations ± SD for each moment of time) obtained from each species, respectively. Baboon and rhesus macaque groups demonstrated TG curve ranges that most closely approximated humans. Swine and rat TG curves did not differ from each other (Fig. 1), but swine plasma was not able to generate plasmin at the tPA concentration used in this study (Fig. 2a). Guinea pig plasmas demonstrated extremely low TG and the lowest PG activity of all species evaluated. The plasmin peaks in rats, guinea pigs and some rabbits were reached significantly later than in the samples of human and non-human primates.

### Two types of rabbit PG curves

**Figure 3:**
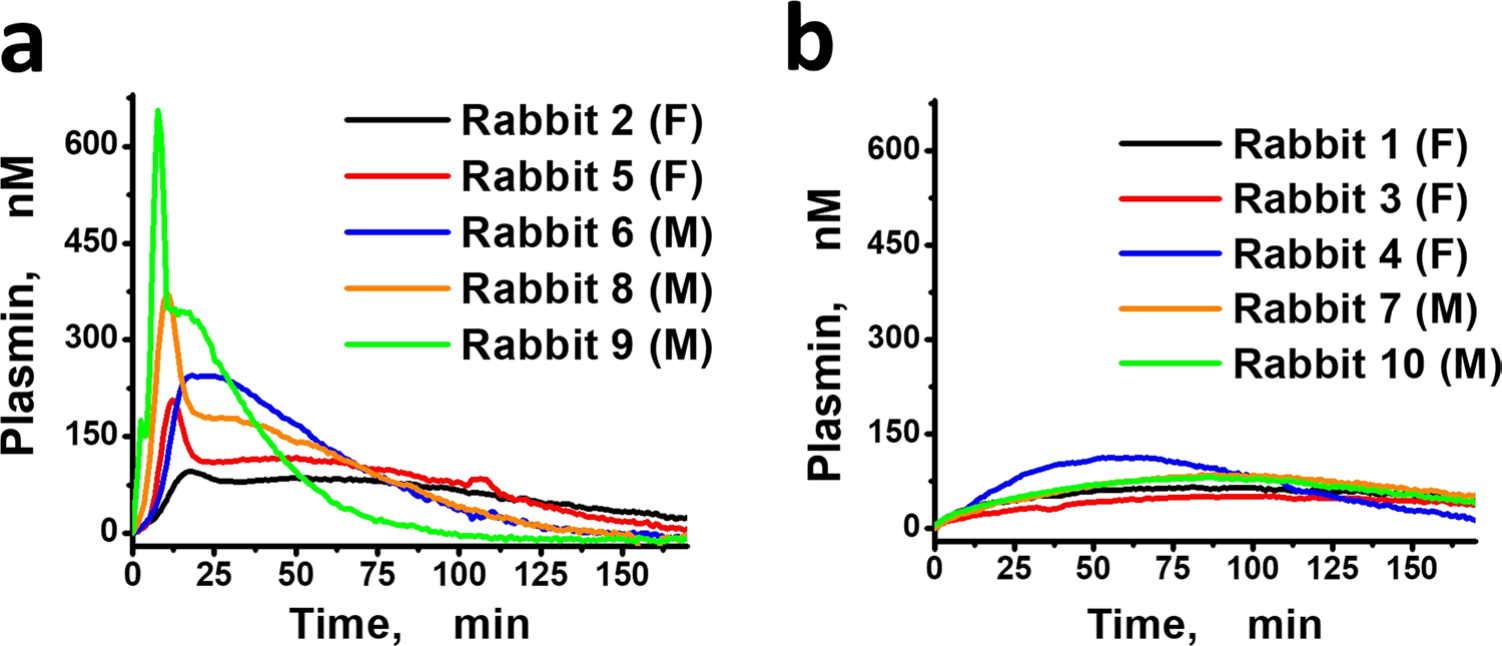
The two different types of PG curves obtained from rabbit plasma samples. (**a**) The two-peaked PG curves with high production rate (range 6.21–85.36 nM/min, mean 33.94, SD 31.95). (**b**) The PG curves with low production rate (range 0.65–2.65 nM/min, mean 1.32, SD 0.79). M means smple from male, F means sample from female.

In Fig. 2b rabbit PG curves demonstrated large within species variability compared to other species. Two types of PG responses occurred upon tPA addition to rabbit plasmas. The first was characterized by high values of PR (6.21–85.36 nM/min) and two-peaked PG curves (Fig. 3a). The PG curves of the second type demonstrated low PR (0.65–2.65 nM/min) (Fig. 3b). The appearance of two types of PG curves in rabbits was independent of male or female sex of the animals.

### STPGA parameters distributions

**Figure 4:**
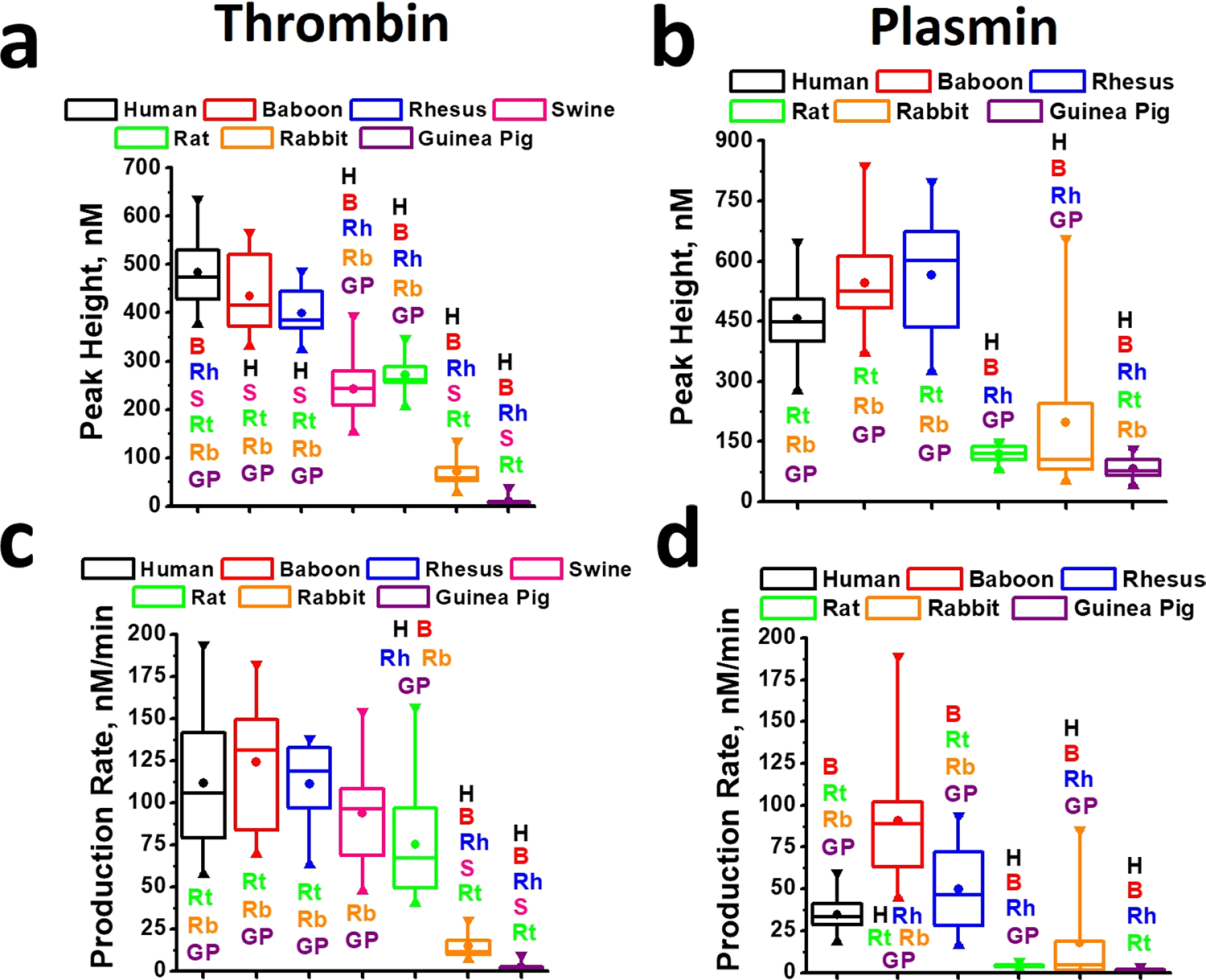
(**a**,**b**) Thrombin (**a**) and plasmin (**b**) PH values obtained in PPPs from PPPs from different animals. (**c**,**d**) Thrombin (**c**) and plasmin (**d**) PR values obtained in PPPs from PPPs from different animals. Upper horizontal line of each box indicates the 75th percentile, and the lower horizontal line of box – indicates the 25th percentile, horizontal line inside box – median, circle inside box – mean value, ▼ – maximal value, ▲ – minimal value. H, B, Rh, S, Rt, Rb and G indicate significant difference from human samples (H), baboon samples (B), rhesus samples (Rh) swine samples (S), rat samples (Rt), rabbit samples (Rb) and Guinea pig samples (G). Statistics was obtained by Kruskal-Wallis test. Significance was set at a p-value less than 0.05.

The peak height (PH) and Production Rate (PR) values were calculated as the main parameters of the STPGA curves as previously reported^26^. Figure 4 shows box-plots of the distributions of the PH and PR values between all the species evaluated in the study. Tables 1 and 2 show the mean, median and coefficient of variance (CV) of TG and PG parameters, respectively. The values of STPGA parameters obtained from each species are shown in Tables S1–S7. Thrombin, as well as plasmin, PH and PR values from primates were significantly different from other species except swine thrombin PR values. STPGA parameter values from humans, baboons and rhesus macaues were most similar compared to other species, nonetheless, some of PH and PR values differed amongst primates. Human thrombin PH values were significantly greater than those from other primates, while their PH values did not differ between each other (Fig. 4a). Additionally, plasmin PR from baboon plasmas was significantly greater than rhesus macaques (Fig. 4d). Thrombin PH and PR values did not differ between rat and swine plasmas (Fig. 4a,c). Rabbit and Guinea pig TG parameters were the lowest observed in the study and differed significantly from each other and other species (Fig. 4a,c). Because of the large variability caused by the two types of responses in rabbit PG parameters (Fig. 3), almost no differences from rat or guinea pig were detected. However, PG parameters from primates demonstrated a significant difference from rabbits (Fig. 4b,d). Guinea pig plasmin PR and PR values were the lowest in the study and significantly differed from all other species except the rabbits PH values (Fig. 4b,d).

**Table 1:** The mean, median, SD and coefficient of variance (CV) values for TG parameters from different species.

Species	Thrombin Concentration Peak				Thrombin Production Rate			
	Mean, nM	Median, nM	SD, nM	CV, %	Mean, nM/min	Median, nM/min	SD, nM/min	CV, %
Human	484.25	473.47	69.20	14.03	111.51	105.70	37.85	33.33
Baboon	433.78	415.78	80.49	17.60	124.22	131.18	38.09	29.09
Rhesus	398.95	384.26	50.27	12.06	111.22	118.65	25.27	21.75
Swine	242.35	242.85	56.85	22.86	93.60	96.31	29.72	30.95
Rat	270.28	261.21	37.11	13.38	75.19	67.16	30.57	39.63
Rabbit	71.60	58.15	35.64	47.23	14.93	11.15	8.73	55.49
Guinea pig	9.86	7.28	9.96	96.99	2.51	1.62	2.85	109.20

**Table 2:** The mean, median, SD and coefficient of variance (CV) values for PG parameters from different species.

Species	Plasmin Concentration Peak				Plasmin Production Rate			
	Mean, nM	Median, nM	SD, nM	CV, %	Mean, nM/min	Median, nM/min	SD, nM/min	CV, %
Human	457.20	448.23	77.23	16.59	34.80	33.32	10.00	28.23
Baboon	546.26	524.69	127.39	22.12	90.81	88.93	40.83	42.66
Rhesus	565.84	601.45	157.37	26.63	49.78	46.35	26.12	50.24
Swine	**Not Detected PG**				**Not Detected PG**			
Rat	186.32	182.82	21.55	16.93	6.45	6.43	1.00	47.79
Rabbit	197.71	104.72	189.99	91.17	17.63	4.43	27.38	147.34
Guinea pig	81.98	76.41	26.24	30.75	1.48	1.19	0.87	56.79

### Correlations between PH and PR values in different species

TG and PG are affected by various promoting (prothrombin, plasminogen, factor V and VIII, etc.) and inhibiting (antithrombin-III, α_2_-macroglobulin, α_2_-antiplasmin etc.) factors present in plasma. Any one of these factors can affect PH or PR values. Thus, the relations between STPGA parameters can reflect the balance between all the factors that affect TG and PG and their distribution among individuals. Since the concentrations of these factors can be variable across different species, the correlations between PH and PR values can be species-dependent. Table 3 shows the correlations between thrombin and plasmin PH and PR values in each species. In human samples the thrombin PH and PR values showed a significant correlation, however, the plasmin PH and PR values did not correlate. Opposite effects were observed in baboons where no correlation between thrombin PH and PR occurred, while significant correlation between PG parameters were detected. Rhesus macaque samples demonstrated correlations in both TG and PG parameters. In rat plasmas, TG parameters did not correlate while plasmin PH significantly correlated to plasmin PR. In rabbits, significant correlations in both TG and PG parameters were observed. Guinea pig samples showed significant correlation between TG and PG parameters as well. In swine, thrombin PH and PR values also significantly correlated. PG parameter correlations were not compared for swine due to the absence of PG in this species at the tPa concentrations used in this study. Finally, the correlations between coagulation (TG) and fibrinolysis (PG) parameters were generally weak for all the species evaluated (data not shown).

**Table 3:** Correlations between PH and PR parameters in TG and PG. Pearson’s correlation coefficients (R) and p-values are presented.

Species	Thrombin Generation		Plasmin Generation	
	R	p	R	p
Human, N = 28	0.7	<0.001	−0.37	0.85
Baboon, N = 10	0.37	0.29	0.84	0.0022
Rhesus, N = 12	0.71	0.01	0.96	<0.001
Swine, N = 20	0.87	<0.001	Not Detected PG	
Rat, N = 20	−0.33	0.16	0.82	<0.001
Rabbit, N = 10	0.96	<0.001	0.99	<0.001
Guinea pig, N = 14	0.97	<0.001	0.61	0.025

## Discussion

The data present herein suggests that TG and PG parameters can differ by more than 10 times across a range of species from rodents to humans. This indicates that the regulation of hemostasis differs substantially among species. Our STPGA assay results suggest that non-human primate species (baboon and rhesus macaque) demonstrated TG and PG that approximated humans more closely than rodents, rabbits or swine. Nonetheless, within the most comparable species (humans, baboons and rhesus macaques), thrombin and plasmin PH and PR associations were uniquely different (Table 3). This data suggests that significant interspecies difference exists among individuals with regards to distributions of pro- and anti-coagulants as well as pro- and anti-fibrinolytic factors that affect TG and PG. We show that human fibrinolysis is different from all other species because only human plasmin PH and PR values did not show any association. Our rat and baboon TG data demonstrated the similar absence of association between individual PH and PR values. Another interesting observation was the two differing PG responses in rabbits, which was not observed in rabbit TG. This observation appeared not to be just high diversity, but two distinctly different fibrinolytic system responses. We could not attribute this effect to males or females or to any other identifiable trait of the animals. Nonetheless, the effect could result from interplay between distributions of different pro- and antifibrinolytic agents among individual rabbits and is a relevant subject for future studies. While the total number of rabbits evaluated were relatively low, the production rates of the first grouping of rabbits (Fig. 3a) are at least an order of magnitude higher than the second grouping (Fig. 3b). Furthermore, the shape of the plasmin generation curves between these two groups qualitatively varies from two-peaked (Fig. 3a) to one-peaked (Fig. 3b) and the values of the production rates between these two groups differ significantly. Taken together, this data suggests that there is a probability of two different regimes of plasmin generation in rabbits based on the very distinct response groupings. In our study, we show that despite statistical equality between swine and rat TG, swine plasmas demonstrated strong resistance to tPA indicating a relevant difference in fibrinolysis and overall hemostasis in this species.

Studies that have investigated TG within the context of species comparison and human translation are limited. Two studies since 2008 have performed TG comparisons to humans in more than two different animals^6,10^. Data from the study by Bel at al. in baboon and swine compared results to humans and is consistent with our data on TG^6^. TG studies on baboon blood^27,28^ and on the blood of other species^10^ did not evaluate TG and PG in the same sample as performed in our STPGA assay. Despite methodological differences, species comparisons in TG in the study by Siller-Matula *et al*. were similar to our results^10^. Human PH values were larger than in rat, swine and rabbit, while guinea pig PH values were the smallest. Rat and rabbit are commonly used species to study TG^3–5,9,29–32^. Among these studies, several used TF concentrations consistent with our study, and subsequently PH values were also similar to those presented in our study^3,4,9,29,30^. Up to this time, there are no studies that report TG in rhesus macaque or Guinea pig, despite both species being used as animal models in hemostasis research^33–38^. It is known that guinea pig blood demonstrates low activity of factors VII and X, as well as prothrombin compared to humans^17,39,40^, which may explain the low level of TG in guinea pigs. The low TG PH values in rats and rabbits can also be explained by low plasma concentrations of factor X comparing to humans^17,40^. Results from the swine TG assay are supported by known elevation of factors V, VIII and IX, and a lack of both factor VII and prothrombin, which can lead to the observation of lower PH values, comparable the rat^17^. Thus, the present study suggests that inter-species animal coagulation activity can differ from humans based on differences in coagulation factors that are more critical to thrombin generation. Considering that there is a sparsity of data on TG performed by the same assay across a range of species from rats to humans, the TG data obtained in our study is consistent with the available literature translating animal to human hemostasis.

Although PG has not been performed to understand species differences in fibrinolysis, it is still possible to compare our PG results with those previously published. Most fibrinolysis assays performed on blood samples from different animal species are based on clot lysis time measurements and demonstrate large intra-species variability^8,9,11^. Nonetheless, some of our results can be compared to and are consistent with previously published studies where fibrinolysis was induced by tPA. For example, under our conditions, swine samples were not able to generate plasmin, is supported by existing studies^8,41^. Based on data from these studies, porcine plasminogen is more resistant to both human and porcine tPA than is human plasminogen, which results in lower rates of fibrinolysis in swine. Based on the work of Jankun *et al*.^11^, tPA-induced fibrinolysis in rat blood, measured by TEG was significantly slower than in human blood samples. In the same study, the TEG clot lysis parameter LY30 obtained in rabbit blood samples was much more variable when compared with human and rat, suggesting the potential for two types of PG that were described in our study (Fig. 4). This observation is in line with the large intraspecies variability in fibrinolysis in rabbit blood that was previously published^9,42^. The thromboelastographic parameters that were observed in these studies, demonstrated large intra-species deviations, without further investigation into this species-specific effect. Our STPGA assay further suggests that these observations are real with regard to rabbit fibrinolysis. It is not likely that intraspecies difference in the concentrations of fibrinolytic agents or the inherent variability of elasticity-based experimental methods represent the only explanations for this observation. Two distinct processes of plasmin generation in rabbit remain a distinct possibility. To investigate this observation further and more in-depth, studies focused on rabbit fibrinolysis using PG assays with both human and rabbit tPA should be evaluated across a large number of animals of differing rabbit strains.

To date, baboon, rhesus macaque and guinea pig have not been evaluated in tPA-induced fibrinolysis assays, thus the present study is the first to inform about PG in these species and compare results to human response. Data from fibrinolysis assays is often difficult to interpret based on high variability in results^8,9,11^, while the STPGA assay used in our study enhances the ability to interpret meaningful interspecies differences based on lower intraspecies variation in results (Table 2).

Our study specifically uses human TF and tPA for TG and PG measurements in distinct species samples and this presents a possible limitation. However, several studies do report homology across species with regards to coagulation. For example, the rate of TF complex formation with factor VIIa (FVIIa) as well as activation of plasminogen by tPA can vary among different species, thus presenting limitation to hemostasis comparisons across species, in general. Based on the existing literature, human, rabbit and rat TF and FVIIa are compatible^43^. Data also suggests that rabbit plasma exhibits comparable procoagulant activity following either human or rabbit TF addition^44^. Similarly, surface plasmon resonance data comparing rat-human TF-FVIIa binding has also been reported^45^. Interestingly, guinea pigs (the species with the lowest measured TG parameters) show compatibility with human TF^38^. Finally, plasminogen from several species also seems to be compatible with human tPA^5,41,42^. Current data suggests that interspecies incompatibility in TF and tPA can contribute to small changes in hemostasis assays, nonetheless the overall comparison of species studied here and compared with humans, reflects similarities and differences in species hemostasis based on our STPGA assay.

Based on the results, we can suggest the following conclusions:

1. Our results provide relevant data on species-dependent blood coagulation and fibrinolysis in a simultaneous TG and PG assay that is conducted on the same plasma sample.
2. Our data reveals new observations that even within the most similar species (baboon, rhesus macaque and human), differences in coagulation can be detected by associations with thrombin and plasmin generation PH and PR parameters.
3. Simultaneous TG and PG measurements may be useful for understanding differences in hemostasis in humans and across a range of species.

## Materials and Methods

### Study approvals

For animal (Guinea pig, Rhesus macaques and Anubis baboon) plasmas that were not purchased from a licensed vendor, FDA Institutional Animal Care and Use Committee Approval (IACUC) was obtained for the purposes of blood collections. For blood collections from these species, procedures were performed in the Association for Assessment and Accreditation for Laboratory Animal Care International (AAALAC) accredited FDA-White Oak (WO) vivarium under strict adherence to National Institutes of Health guidelines on the care and use of animals. Approved FDA-WO protocols were obtained for donor blood collection from guinea pigs (FDA-WO-IACUC protocol #2018-06) and for non-human primate (Rhesus macaques and Anubis baboons) blood collections (FDA-WO-IACUC protocol #2018-31). All other species (New Zealand White rabbits, Yorkshire swine and Sprague Dawley rats) plasmas were purchased from approved vendors (Innovative Research (Novi, MI, USA) and BioChemed Services (Winchester, VA, USA), under their individual IACUC approval process with strict adherence to National Institutes of Health guidelines on the care and use of animals. Details of procedures for blood collection from Guinea pig, Rhesus macaques and Anubis baboons are described in the plasma samples section detailed below. The purchase of human plasma for the present studies was reviewed by the FDA Research Involving Human Subjects Committee (RIHSC) under protocol #18297-115044009 and deemed not to require RIHSC approval because it does not meet the requirements of research involving human subjects as defined in the US code of regulations (45 CFR 46).

### Plasma samples

All samples evaluated were obtained from individual human and animal donors. Commercially available citrated PPP from 28 humans and 10 New Zealand White rabbits was purchased from Innovative Research (Novi, MI, USA). Citrated PPP from 20 Yorkshire Swine and 20 Sprague Dawley rats was purchased from BioChemed Services (Winchester, VA, USA). Citrated PPP was obtained from femoral vein of 12 Rhesus macaques, and 10 Anubis (Olive) baboons. Blood collections from Rhesus macaques and Anubis baboons were approved under FDA-WO-IACUC protocol #2018-31. Collections were performed to obtain 20 ml of whole blood (<10% of animals blood volume), collected into a syringe containing citrate phosphate dextrose using a 20 G needle from the femoral vein, while animals were under ketamine/dexmedetomidine (7 mg/kg/0.2 mg/kg) anesthesia. Hartley Blood collections from Guinea pigs were approved under FDA-WO-IACUC protocol #2018-06. Blood was obtained from ketamine/xylazine HCl (100 mg/kg/5 mg/kg) anesthetized guinea pigs from an implanted carotid artery catheter using a 5 ml syringe containing citrate phosphate dextrose. Whole blood from Rhesus macaques, Anubis baboons and Guinea pigs was centrifugated for 15 min at 1500 g to obtain PPP. An equal number of male and female donors were used in the experiments. PPP samples were stored at −80 °C. Before experiment, samples were thawed and incubated for 1 hour under 37 °C.

### Simultaneous thrombin plasmin generation assay (STPGA)

STPGA was performed as previously described^21^. Briefly, PPP samples premixed with thrombin specific substrate Z-Gly-Gly-Arg-AMC (Bachem, Bubendorf, Switzerland) or plasmin specific substrate Boc- Glu-Lys-Lys-AMC (Bachem, Bubendorf, Switzerland) were induced by activator containing CaCl_2_ (final assay concentration 16 mM, Sigma-Aldrich, St. Louis, MO, USA), PPP-reagent (TF and phospholipids for Calibrated Automated Thrombography, Diagnostica Stago, Asnières sur Seine, France), and recombinant human tPA (MyBioSource, San-Diego, CA, USA). The final concentration of TF was 4.5 pm, the final concentration of tPA was 0.7 µg/ml.

### Statistical analysis

For interspecies STPGA parameters comparison a Kruskal-Wallis test for 7 independent groups of samples was used. Between group differences were significant if a p-value of less than 0.05 was observed. To obtain correlations between PH and PR values the Pearson’s correlation coefficient and level of significance were calculated. Similarly, significance was set at a p-value less than 0.05.

## Supplementary information

Supplementary material.
