# Supplementary material for: Interspecies comparison of simultaneous thrombin and plasmin generation
Source: Sci Rep. 2020 Mar 3;10:3885. doi: 10.1038/s41598-020-60436-1 (PMC7054422; doi:10.1038/s41598-020-60436-1)
Supplement: Supplementary file 1 — Supplementary material. [file 41598_2020_60436_MOESM1_ESM.docx]

**Interspecies comparison of simultaneous thrombin and plasmin generation**

Ivan D. Tarandovskiy, Hye Kyung H. Shin, Jin Hyen Baek, Elena Karnaukhova, Paul W. Buehler

***Supplementary Material***

Table S1. Thrombin and plasmin concentration peak and production rate values obtained in PPPs from healthy human plasma samples.

| Human | | | | |
| --- | --- | --- | --- | --- |
| Donor | Thrombin | | Plasmin | |
|  | Peak Height, nM | Production Rate, nM/min | Peak Height, nM | Production Rate, nM/min |
| 1 | 427.05 | 106.20 | 465.55 | 29.00 |
| 2 | 537.44 | 104.54 | 483.51 | 27.22 |
| 3 | 568.58 | 154.59 | 423.53 | 31.27 |
| 4 | 559.10 | 159.82 | 373.42 | 34.24 |
| 5 | 508.29 | 193.88 | 468.55 | 30.65 |
| 6 | 620.74 | 181.34 | 465.01 | 18.65 |
| 7 | 636.21 | 153.26 | 648.30 | 42.08 |
| 8 | 504.92 | 99.91 | 431.48 | 29.36 |
| 9 | 415.87 | 81.20 | 452.08 | 49.49 |
| 10 | 571.48 | 128.47 | 435.63 | 46.85 |
| 11 | 512.91 | 109.69 | 396.61 | 50.02 |
| 12 | 452.00 | 69.37 | 367.92 | 31.57 |
| 13 | 449.90 | 74.04 | 533.26 | 46.16 |
| 14 | 423.85 | 57.62 | 444.38 | 32.23 |
| 15 | 556.64 | 108.06 | 471.23 | 35.45 |
| 16 | 523.63 | 96.16 | 403.67 | 59.93 |
| 17 | 377.04 | 74.16 | 566.67 | 36.53 |
| 18 | 505.10 | 117.96 | 396.33 | 26.84 |
| 19 | 386.10 | 77.80 | 543.98 | 40.14 |
| 20 | 429.90 | 80.50 | 396.00 | 37.73 |
| 21 | 470.05 | 149.58 | 505.26 | 22.76 |
| 22 | 476.89 | 162.49 | 592.42 | 20.80 |
| 23 | 497.67 | 132.98 | 429.43 | 36.03 |
| 24 | 427.79 | 105.20 | 503.80 | 19.07 |
| 25 | 464.89 | 125.33 | 521.91 | 45.74 |
| 26 | 419.38 | 90.95 | 277.56 | 32.54 |
| 27 | 432.82 | 68.71 | 418.94 | 34.09 |
| 28 | 402.79 | 58.58 | 385.10 | 27.89 |

Table S2. Thrombin and plasmin concentration peak and production rate values obtained in PPPs from rhesus monkey plasma samples.

| Baboon | | | | |
| --- | --- | --- | --- | --- |
| Donor | Thrombin | | Plasmin | |
|  | Peak Height, nM | Production Rate, nM/min | Peak Height, nM | Production Rate, nM/min |
| 1 | 450.07 | 149.19 | 612.32 | 76.40 |
| 2 | 567.27 | 147.17 | 482.68 | 95.74 |
| 3 | 520.51 | 102.31 | 622.26 | 109.49 |
| 4 | 374.53 | 182.23 | 521.23 | 44.81 |
| 5 | 412.13 | 83.93 | 515.18 | 101.90 |
| 6 | 419.43 | 129.52 | 371.74 | 63.42 |
| 7 | 362.07 | 132.85 | 429.11 | 49.25 |
| 8 | 372.38 | 82.10 | 540.86 | 92.00 |
| 9 | 528.61 | 163.58 | 839.08 | 189.20 |
| 10 | 330.78 | 69.35 | 528.14 | 85.86 |

Table S3. Thrombin and plasmin concentration peak and production rate values obtained in PPPs from baboon plasma samples.

| Rhesus Monkey | | | | |
| --- | --- | --- | --- | --- |
| Donor | Thrombin | | Plasmin | |
|  | Peak Height, nM | Production Rate, nM/min | Peak Height, nM | Production Rate, nM/min |
| 1 | 347.46 | 63.39 | 670.47 | 64.30 |
| 2 | 396.05 | 120.97 | 524.26 | 44.43 |
| 3 | 384.45 | 70.64 | 471.56 | 36.33 |
| 4 | 433.81 | 126.99 | 591.40 | 48.27 |
| 5 | 361.41 | 93.63 | 399.18 | 19.48 |
| 6 | 463.71 | 135.39 | 678.58 | 81.91 |
| 7 | 487.26 | 138.13 | 338.41 | 16.44 |
| 8 | 384.08 | 134.00 | 798.33 | 93.59 |
| 9 | 374.84 | 116.32 | 611.51 | 39.53 |
| 10 | 323.96 | 100.21 | 611.51 | 55.54 |
| 11 | 374.24 | 103.63 | 770.04 | 80.06 |
| 12 | 456.17 | 131.36 | 324.85 | 17.44 |

Table S4. Thrombin and plasmin concentration peak and production rate values obtained in PPPs from swine plasma samples.

| Swine | | |
| --- | --- | --- |
| Donor | Thrombin | |
|  | Peak Height, nM | Production Rate, nM/min |
| 1 | 229.09 | 106.87 |
| 2 | 280.66 | 136.84 |
| 3 | 248.90 | 105.17 |
| 4 | 206.53 | 66.37 |
| 5 | 154.95 | 47.53 |
| 6 | 153.64 | 59.73 |
| 7 | 210.40 | 72.93 |
| 8 | 306.32 | 119.09 |
| 9 | 218.55 | 101.60 |
| 10 | 244.53 | 76.89 |
| 11 | 260.61 | 83.02 |
| 12 | 296.74 | 109.77 |
| 13 | 278.25 | 105.17 |
| 14 | 278.29 | 141.23 |
| 15 | 208.75 | 58.56 |
| 16 | 264.30 | 101.37 |
| 17 | 394.43 | 154.25 |
| 18 | 241.16 | 91.24 |
| 19 | 194.84 | 70.73 |
| 20 | 176.15 | 63.67 |

Table S5. Thrombin and plasmin concentration peak and production rate values obtained in PPPs from rat plasma samples.

| Rat | | | | |
| --- | --- | --- | --- | --- |
| Donor | Thrombin | | Plasmin | |
|  | Peak Height, nM | Production Rate, nM/min | Peak Height, nM | Production Rate, nM/min |
| 1 | 256.93 | 44.81 | 132.52 | 4.06 |
| 2 | 273.67 | 40.58 | 191.53 | 7.41 |
| 3 | 220.68 | 110.62 | 254.57 | 9.24 |
| 4 | 258.74 | 96.51 | 207.77 | 6.84 |
| 5 | 273.32 | 75.20 | 213.40 | 13.10 |
| 6 | 259.16 | 63.66 | 157.22 | 6.51 |
| 7 | 306.68 | 49.21 | 167.21 | 2.62 |
| 8 | 347.74 | 56.97 | 182.92 | 6.35 |
| 9 | 220.68 | 110.62 | 254.57 | 9.24 |
| 10 | 258.74 | 96.51 | 207.77 | 6.84 |
| 11 | 273.32 | 75.20 | 213.40 | 13.10 |
| 12 | 259.16 | 63.66 | 157.22 | 6.51 |
| 13 | 306.68 | 49.21 | 167.21 | 2.62 |
| 14 | 347.74 | 56.97 | 182.92 | 6.35 |
| 15 | 206.46 | 102.84 | 160.70 | 9.64 |
| 16 | 250.78 | 97.30 | 205.20 | 4.87 |
| 17 | 302.60 | 156.55 | 169.41 | 4.29 |
| 18 | 263.26 | 70.65 | 162.89 | 3.39 |
| 19 | 252.65 | 46.48 | 182.71 | 2.83 |
| 20 | 266.64 | 40.25 | 155.25 | 3.27 |

Table S6. Thrombin and plasmin concentration peak and production rate values obtained in PPPs from rabbit plasma samples.

| Rabbit | | | | |
| --- | --- | --- | --- | --- |
| Donor | Thrombin | | Plasmin | |
|  | Peak Height, nM | Production Rate, nM/min | Peak Height, nM | Production Rate, nM/min |
| 1 | 58.49 | 12.29 | 66.69 | 0.81 |
| 2 | 78.60 | 18.14 | 96.44 | 6.21 |
| 3 | 132.38 | 30.51 | 51.98 | 0.65 |
| 4 | 57.82 | 8.41 | 113.01 | 2.65 |
| 5 | 54.54 | 9.76 | 207.25 | 18.65 |
| 6 | 26.94 | 6.91 | 244.41 | 15.57 |
| 7 | 134.40 | 30.54 | 86.36 | 1.16 |
| 8 | 52.08 | 11.58 | 372.56 | 43.91 |
| 9 | 75.66 | 10.44 | 656.41 | 85.36 |
| 10 | 45.09 | 10.72 | 81.96 | 1.31 |

Table S7. Thrombin and plasmin concentration peak and production rate values obtained in PPPs from guinea pig plasma samples.

| Guinea Pig | | | | |
| --- | --- | --- | --- | --- |
| Donor | Thrombin | | Plasmin | |
|  | Peak Height, nM | Production Rate, nM/min | Peak Height, nM | Production Rate, nM/min |
| 1 | 9.03 | 2.70 | 76.41 | 1.19 |
| 2 | 0.00 | 0.00 | 91.47 | 2.92 |
| 3 | 7.28 | 2.52 | 79.85 | 1.47 |
| 4 | 37.46 | 9.72 | 40.67 | 0.69 |
| 5 | 6.80 | 1.86 | 112.72 | 1.49 |
| 6 | 0.00 | 0.00 | 105.44 | 3.59 |
| 7 | 6.02 | 1.42 | 55.92 | 0.76 |
| 8 | 9.04 | 3.25 | 64.64 | 1.12 |
| 10 | 8.23 | 0.98 | 131.26 | 1.70 |
| 11 | 9.60 | 1.62 | 69.88 | 0.88 |
| 12 | 5.16 | 0.73 | 75.96 | 1.06 |
| 13 | 6.81 | 0.71 | 106.66 | 1.63 |
| 14 | 22.79 | 7.15 | 54.86 | 0.67 |
